# The long‐term outcome of patients in the LRF CLL4 trial: the effect of salvage treatment and biological markers in those surviving 10 years

**DOI:** 10.1111/bjh.13824

**Published:** 2015-10-12

**Authors:** Monica Else, Rachel Wade, David Oscier, Daniel Catovsky

**Affiliations:** ^1^ Division of Molecular PathologyThe Institute of Cancer ResearchLondonUK; ^2^ Clinical Trial Service UnitOxfordUK; ^3^ Department of Molecular PathologyRoyal Bournemouth HospitalBournemouthUK

**Keywords:** Chronic lymphocytic leukaemia, CLL, prognostic factors, salvage therapy, survival, clinical trials

## Abstract

With 10+ years follow‐up in the Leukaemia Research Fund (LRF) CLL4 trial, we report the effect of salvage therapy, and the clinical/biological features of the 10‐year survivors treated for chronic lymphocytic leukaemia (CLL). Overall survival (OS) was similar in the three randomized arms. With fludarabine‐plus‐cyclophosphamide (FC), progression‐free survival (PFS) was significantly longer (*P* < 0·0001), but OS after progression significantly shorter, than in the chlorambucil or fludarabine arms (*P* < 0·0001). 614/777 patients progressed; 524 received second‐line and 260 third‐line therapy, with significantly better complete remission (CR) rates compared to first‐line in the chlorambucil arm (7% vs. 13% after second‐, 18% after third‐line), but worse in the FC arm (38% vs. 15% after both second and third‐line). OS 10 years after progression was better after a second‐line CR *versus* a partial response (36% vs. 16%) and better with FC‐based second‐line therapy (including rituximab in 20%) or a stem cell transplant (28%) *versus* all other treatments (10%, *P* < 0·0001). The 176 (24%) 10‐year survivors tended to be aged <70 years, with a “good risk” prognostic profile, stage A‐progressive, achieving at least one CR, with a first‐line PFS >3 years and receiving ≤2 lines of treatment. In conclusion, clinical/biological features and salvage treatments both influence the long‐term outcome. Second‐line therapies that induce a CR can improve OS in CLL patients.

Long survival in patients with chronic lymphocytic leukaemia (CLL) who require treatment depends on a variety of factors, which include age, the absence of co‐morbidities, disease biology and the efficacy and toxicity of both initial and subsequent therapies. Over the last decade, clinical trials of first‐line therapy have demonstrated improved response rates and longer progression‐free survival (PFS) with the chemotherapy combination fludarabine plus cyclophosphamide (FC), and further improvements in response rates and PFS, as well as longer overall survival (OS), with the addition of rituximab to this combination (FCR) (Keating *et al*, 2005; Hallek *et al*, 2010; Böttcher *et al*, 2012). The most recent trials present the prospect of still better outcomes with the introduction of new effective treatments, including antibodies, *BCL2* antagonists and BCR kinase inhibitors, given both in untreated and pre‐treated patients (Souers *et al*, 2013; Byrd *et al*, 2014; Furman *et al*, 2014; Goede *et al*, 2014).

Despite the excitement generated by novel therapies, the longer‐term follow‐up available from the earlier chemotherapy and chemo‐immunotherapy trials provides an opportunity to explore the contribution of salvage therapies to long‐term outcomes. Few such studies have been published. Woyach *et al* (2011) reported on 104 patients randomly assigned to receive fludarabine with rituximab, either concurrently or sequentially, with a median follow‐up of 10 years (range 5½–11 years). Tam *et al* (2014) reported on 300 patients treated initially with FCR, with a median follow‐up of 12 years (range 6–14 years).

The Leukaemia Research Fund (LRF) CLL4 trial randomized 777 patients to receive chlorambucil or fludarabine alone, or the combination FC, and showed no OS advantage for any of these first‐line treatments, even though a significantly better response rate and longer PFS were seen after FC (Catovsky *et al*, 2007). The trial now has more than 10 years follow‐up on all surviving patients (median 12 years, range 10–16 years) and, to our knowledge, this report is the largest and the longest‐term so far published from a randomized trial. It includes an analysis of how second‐ and third‐line treatments might have contributed to the similarity of the survival curves across the three randomized treatment arms. Moreover, while the baseline prognostic factors for patients with CLL have been well studied and documented in this trial (Oscier *et al*, 2010, 2013; Gonzalez *et al*, 2011, 2013), the biological and treatment factors associated with patients who survive 10 years or more have not been previously reported. We include here 176 such patients with baseline biological, cytogenetic and molecular data and documented treatment histories, allowing us to consider the characteristics of these long‐term survivors.

## Patients and methods

In the LRF CLL4 trial, 777 patients were randomized between February 1999 and October 2004 to receive chlorambucil, fludarabine or FC. The patients were previously untreated, 191 (25%) having Binet stage A‐progressive disease, 352 (45%) stage B and 234 (30%) stage C. The male:female ratio was 3:1 and the median age was 65 years (range 35–86 years). Biological, cytogenetic and molecular markers were available and have been reported elsewhere, together with a description of the cut‐offs used to define positivity (Oscier *et al*, 2010, 2013). These markers were available at randomization only, not at relapse. Risk groups, which were evenly distributed across the three randomised arms, were initially identified as follows: poor risk – known *TP53* deletion >10% (17p del by FISH); intermediate risk – without *TP53* deletion (≤10%) and with at least one of: unmutated *IGHV* genes and/or *IGHV3‐21* usage, 11q deletion, β‐2 microglobulin ≥4 mg/l; good risk – none of the above and mutated *IGHV* genes (Oscier *et al*, 2010). Since then, several newer prognostic markers have been investigated in this trial: *TP53*,* SF3B1* and *NOTCH1* mutations, and *CLLU1* expression (Gonzalez *et al*, 2011, 2013; Oscier *et al*, 2013).

After disease progression, a minority of trial patients (*n* = 84) were randomized to receive the results of an *in‐vitro* assay measuring drug resistance, which could be used by the treating physician to guide the choice of second‐line treatment. The results of this randomization have been previously reported (Matutes *et al*, 2013), but here we describe only the treatment given. All other second‐line and all third‐line and subsequent treatments were given according to the treating physician's choice.

Clinical follow‐up, documenting patients' first, second and third‐line treatment history was to 31 October 2010. Follow‐up for OS only was to January 2015 for UK patients (median 11·8 years; range 10·2–15·9 years). The deaths of LRF CLL4 trial patients still resident in the UK were flagged and reported to the Clinical Trial Service Unit at Oxford. For 44 surviving patients resident outside the UK, OS was censored at 31 October 2010, the date these patients were last known to be alive. Thus for 733 patients survival data were available for a minimum of 10·2 years, allowing us to analyse the characteristics of the 10‐year survivors.

Survival was estimated by the Kaplan‐Meier method and compared using the log‐rank test. OS was calculated from randomization to death from any cause. OS after progression was calculated from the date of documented disease progression after first‐line treatment to death from any cause. PFS was time from randomization to relapse needing further treatment, progression or death from any cause. For non‐response (NR) and progressive disease (PD), date of progression was when NR/PD was recorded. Second‐line PFS was calculated from the start of second‐line treatment. We calculated odds ratios (ORs) and 95% confidence intervals (CIs) as exp[(*O*−*E*)/Var ± 1·96/√Var], in which *O* is the observed events, *E* is the expected events, and Var is the variance. A plot of OS after progression by second‐line treatments was drawn to show the relative effect of these treatments within subgroups defined by randomized arm. Within each subgroup, the observed minus expected (O–E) number of events and its variance (Var) are given.

Multivariate analyses were performed by means of stepwise generalized linear modelling. Values of *P* ≤ 0·05 (two sided) were considered significant. Analyses were performed using the STATISTICA software from StatSoft, a subsidiary of Dell Inc. (Bracknell, UK).

The LRF CLL4 trial was registered (ISRCTN58585610) and approved by a UK multicentre research ethics committee. All patients provided written informed consent. The main trial endpoints have been previously reported (Catovsky *et al*, 2007).

## Results

### The patients' journey from first‐line randomization to the final follow‐up

**Figure 1:**
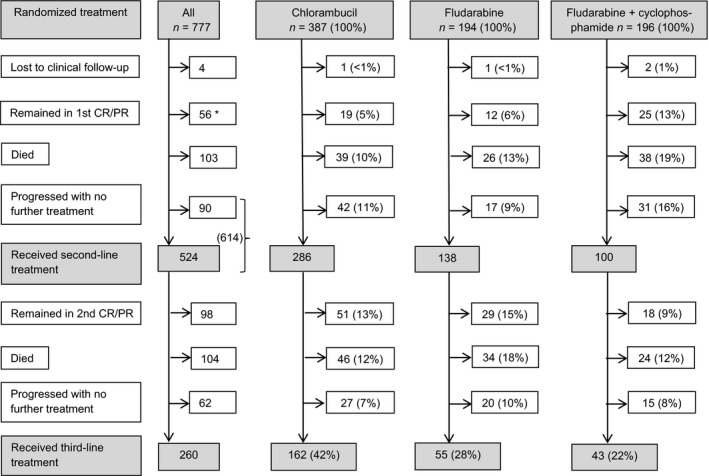
Treatment history of patients in the Leukaemia Research Fund (LRF) chronic lymphocytic leukaemia (CLL)4 trial. Consort diagram showing the treatment history of the 777 trial patients, from randomization until latest follow‐up (censored at 31 October 2010). CR, complete remission; PR, partial remission. *This number included 19% (*n* = 23) of all the trial patients who obtained a CR and 7% (*n* = 33) of those with a PR, including nodular PR.

At the last clinical follow‐up in 2010, at a median of 7·1 years since randomization (range 6·0–11·7 years), 4 patients were lost to follow‐up, while 56 (7%) remained in their first complete or partial remission (CR/PR) (Fig 1). The proportion of patients who remained in first remission was more than twice as high in the FC arm compared to the other two arms: FC 13% vs. chlorambucil 5% {odds ratio [OR] 2·86 (95% CI: 1·53–5·33), *P* = 0·001} or *versus* fludarabine 6% [OR 2·23 (1·09–4·58), *P* = 0·03] and higher in patients in CR (19%) than in PR (5%; *P* < 0·0001). The proportion of good, intermediate and poor risk (*TP53*‐deleted) patients remaining in remission was 17%, 3% and 0% respectively. No patients with *SF3B1* or *TP53* mutations remained in remission.

There were 103 deaths (13%), without documented disease progression (Fig 1). The remaining 614 patients (79%) progressed, of whom 524 (67%) received a second line of treatment. While nearly three‐quarters of patients in the chlorambucil and fludarabine arms received second‐line treatment, only half of the patients in the FC arm did so. After further disease progression, 260 patients (33%) received one or more subsequent lines of treatment. Third‐line treatments were more frequently given to patients in the chlorambucil arm than in the fludarabine and FC arms.

By January 2015, 596 deaths had been reported (81% of assessable patients). The proportion of deaths due to Richter syndrome was similar across the three randomized trial arms (5% both chlorambucil and fludarabine, 6% FC; *P* = 0·9). The primary cause of death, assessable in 580 patients, was recorded as being other than CLL or infection (mainly other cancers and cardiovascular) in 22% of deaths in the chlorambucil arm, 18% fludarabine and 25% FC (*P* = 0·4).

**Figure 2:**
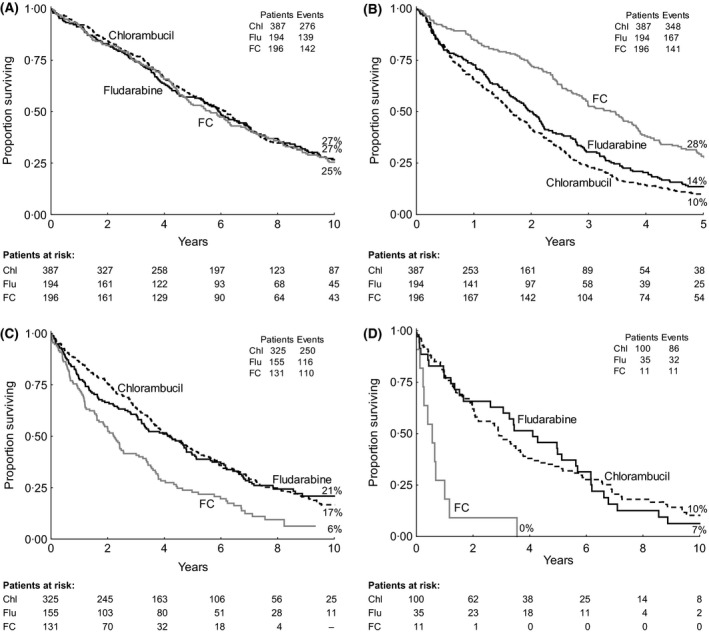
Survival by first‐line randomized treatment. (A) Overall survival (OS) from randomization (*n* = 777). (B) 5‐year progression‐free survival from randomization (*n* = 777). (C) OS after progression in the 614 patients whose disease progressed. (D) OS after progression in non‐responders to first‐line treatment. Of the 109 non‐responders assessable for risk group, the following proportions were poor risk (*TP53*‐deleted): chlorambucil 15%; fludarabine 17%; FC 67%. Chl, chlorambucil; Flu, fludarabine; FC, fludarabine with cyclophosphamide.

At 10 years, OS from randomization was similar in all three randomized arms: 27% (95% CI: 22–31%) with chlorambucil, 27% (20–33%) with fludarabine and 25% (19–32%) with FC (Fig 2A). This was despite the significantly longer PFS with FC (median 40 months) *versus* chlorambucil {median 20 months; hazard ratio [HR] 0·53 (95% CI: 0·44–0·63), *P* < 0·0001} and *versus* fludarabine [median 24 months, HR 0·61 (0·50–0·76), *P* < 0·0001; Fig 2B]. In contrast, for the 614 patients whose disease progressed, OS after progression was correspondingly longer in both the chlorambucil arm [median 49 months, HR 0·56 (0·44–0·70), *P* < 0·0001] and the fludarabine arm [median 51 months, HR 0·57 (0·44–0·75), *P* = 0·0003], compared to FC (median 26 months; Fig 2C). At 10 years after progression, OS was 6% (0–13%) in the FC arm, 17% (12–21%) in the chlorambucil arm and 21% (14–28%) in the fludarabine arm (Fig 2C). When we analysed OS after progression in the non‐responders to first‐line treatment, the 11 patients who were non‐responsive to FC were mostly in the poor risk (*TP53*‐deleted) group and did particularly badly (Fig 2D).

### The results of second‐ and third‐line (salvage) treatments

Second‐line and third‐line treatments included fludarabine as a single agent, FC‐based combination treatments, stem cell transplants (including both autografts and allografts), alemtuzumab, alkylators (mostly chlorambucil) and anthracyclines (mostly in combination with other agents), steroids and other miscellaneous agents (Table 1). Details of these treatments are given in the footnote to Table 1. In the FC‐based combinations, rituximab was included in the combination for 28 patients (20%) at second line and 22 patients (31%) at third line. Stem cell transplants (SCT) were only given to patients aged <65 years at randomization (median 54 years). Alemtuzumab was given to 26% of poor risk (*TP53*‐deleted) patients, but to only 3–4% of patients in the other two risk groups.

**Table 1:** Second and third‐line treatment types and complete response rates

Treatment	Second line		Third line	
	Patients *n* (%)	Complete response rate, %	Patients *n* (%)	Complete response rate, %
Fludarabine as a single agent	138 (26)	10^e^	38 (15)	4^e^
FC‐based combinations^a^	141 (27)	21	71 (27)	21
Stem cell transplant^b^	17 (3)	44	7 (3)	83
Alemtuzumab	29 (6)	38	28 (11)	53
Alkylator or anthracycline^c^	157 (30)	5	67 (26)	8
Steroids & other agents^d^	42 (8)	3	49 (19)	7
TOTAL	524 (100)		260 (100)	

a FC – fludarabine with cyclophosphamide. Combinations included: FC, FC with mitoxantrone (FCM), FC with rituximab (FCR), FCMR; FR (1 patient); pentostatin+C (1 patient).

b Included a total of 8 autografts and 16 allografts, with similar response rates.

c Alkylators – mainly chlorambucil; anthracyclines – mainly CHOP (cyclophosphamide, vincristine, doxorubicin and prednisolone).

d Steroids: high dose methylprednisolone, dexamethasone, prednisolone; other agents included rituximab alone (6 patients), radiotherapy (5 patients), ofatumumab (2 patients), BCL‐2 inhibitors (2 patients).

e All complete responses after fludarabine as a single agent were in the chlorambucil arm.

The best CR rates, at both second and third line, were seen with SCT, including both autografts and allografts, and alemtuzumab (Table 1). FC‐based combinations had CR rates in the middle range. After fludarabine as a second or third‐line single agent, CRs were infrequent and occurred only in the chlorambucil arm. CR rates were also low in the groups including alkylators, anthracyclines, steroids and other agents and were broadly similar, whether the treatments were given at second or third line.

In the chlorambucil and fludarabine arms, the two thirds of patients who received salvage treatment (Fig 1) benefitted from improved CR rates with salvage treatment compared to the first‐line CR rates with the randomized treatment (Table 2). In the chlorambucil arm this improvement was significant both at second‐line (*P* = 0·03) and third‐line (*P* = 0·0007). In the FC arm, when compared to the high response rates seen with first‐line FC, with salvage treatment the CR rates were lower at second line (*P* = 0·0006) and third line (*P* = 0·02).

**Table 2:** Complete response rates after each line of treatment by first‐line randomized arm

Patients	Complete response rate: first‐line (randomized) treatment, %	Complete response rates after the salvage treatments given	
		Second‐line, %	Third‐line, %
Chlorambucil arm	7	13	18
Fludarabine arm	15	17	21
FC arm	38	15	15

FC, fludarabine with cyclophosphamide.

**Figure 3:**
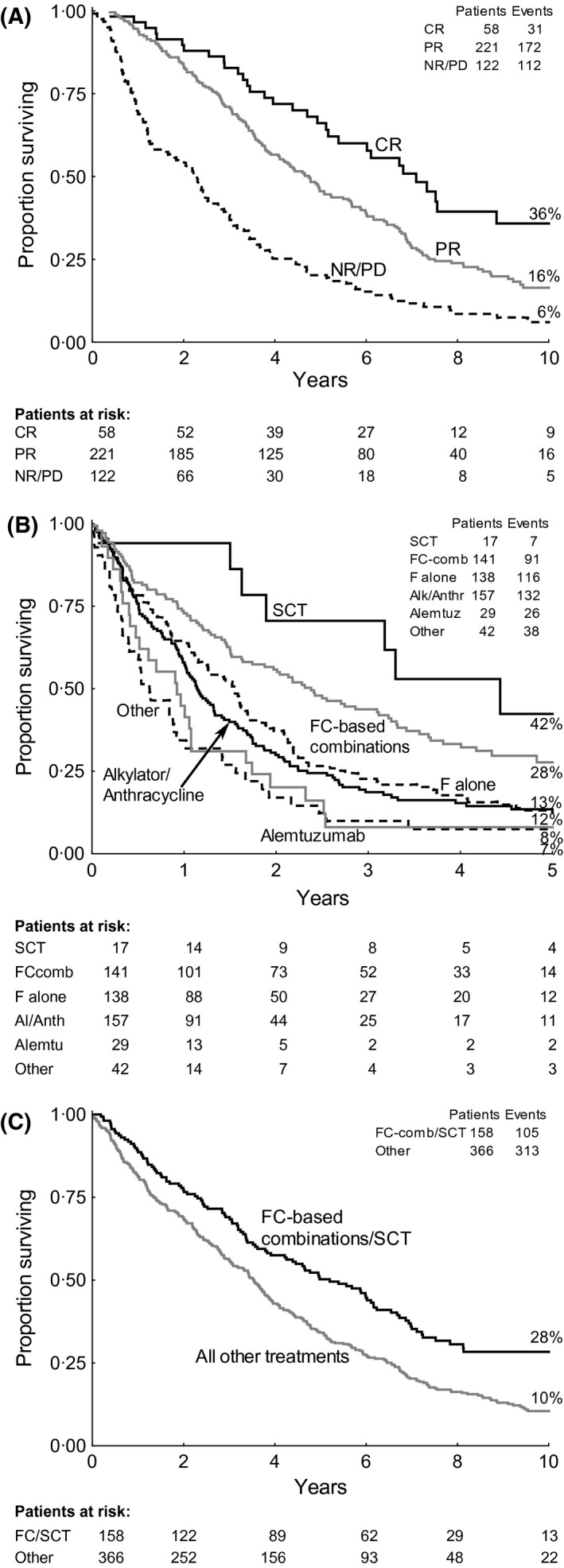
Survival by second‐line treatment. (A) Overall survival (OS) after progression by response to second‐line treatment (401 assessable patients). CR, complete response; PR, partial response; NR/PD, non‐response or progressive disease. (B) 5‐year second‐line progression‐free survival by second‐line treatment type (*n* = 524). SCT, stem‐cell transplant (6 autografts and 11 allografts); F, fludarabine; C, cyclophosphamide; FCcomb, fludarabine with cyclophosphamide based combinations; Al/Anth, alkylator/anthracycline; Alemtu, alemtuzumab. (C) OS after progression by FC‐based combination treatments or SCT *versus* all other treatments (*n* = 524).

Good second‐line responses were associated with better second‐line PFS (not shown) and better OS after progression (Fig 3A). At 10 years after progression 36% (95% CI: 21–51%) of patients who obtained a CR after second‐line treatment were still alive, compared to 16% (11–22%) of those who had a PR and 6% (1–11%) of non‐responders; {CR vs. PR: HR 0·61 [0·42–0·88], *P* = 0·003; PR vs. NR/PD: HR 0·30 (0·20–0·44), *P* < 0·0001}.

Second‐line PFS was significantly better after FC‐based combinations and SCT than after any other form of salvage treatment (Fig 3B). At 5 years, second‐line PFS with FC‐based combinations and SCT grouped together was 29% (21–38) *versus* all other treatments 12% (8–15); HR 0·52 (0·42–0·65), *P* < 0·0001. Although alemtuzumab had good response rates, it had short PFS. We therefore combined FC‐based combinations/SCT, *versus* all other treatments, in the subsequent analyses. FC‐based combinations/SCT were used as second‐line treatments in an equal proportion of patients in the three randomized trial arms (28% chlorambucil, 33% fludarabine, 32% FC, *P* = 0·6).

**Figure 4:**
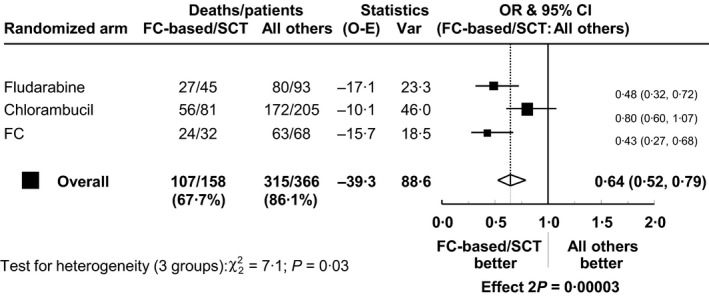
A forest plot showing the relative effect of second‐line treatments on overall survival (OS) after progression within subgroups defined by randomized arm. The odds ratio (OR) is shown as a filled box, with a line indicating the 95% confidence interval (CI). The overall effect after stratification by randomized arm is shown as a diamond whose width indicates the 95% CI of the overall result. The result of the test for heterogeneity is displayed to indicate any evidence of a different effect. FC, fludarabine with cyclophosphamide; FC‐based/SCT, FC‐based combinations or stem cell transplant given as second‐line treatment; all others, all other second‐line treatments.

At 10 years after progression, OS was significantly better in the FC‐based combinations/SCT group [28% (21–36)] *versus* all other treatments {10% [7–14]; HR 0·64 (0·52‐0·79), *P* < 0·0001; Fig 3C}. This difference was particularly marked in patients who had received fludarabine, and especially FC, as their first‐line treatment (Fig 4).

We investigated the effect of second‐line FC‐based combination treatments with (*n* = 28) or without (*n* = 113) rituximab and found that outcomes were better with rituximab: second‐line CR rate 36% (95% CI: 18–54%) vs. 18% (11–25%) respectively (*P* = 0·05); second‐line PFS at 5 years 38% (19–58%) vs. 25% (15–34%; *P* = 0·06); OS 10 years after progression 35% (16–54%) vs. 23% (14–32%; *P* = 0·3).

### The characteristics of the 10‐year survivors (Table 3)

Of the 733 patients with long‐term follow‐up data, 176 patients (24%) survived 10 years from randomization, with an equal proportion in all three randomized trial arms. Women, younger patients and those with Binet disease stage A‐progressive or B were more likely to survive 10 years than men, older patients and those with stage C disease. Over half of the patients classified by prognostic markers as good risk survived 10 years, compared to none of the poor‐risk (*TP53‐*deleted) group. Patients whose first‐line PFS lasted more than 3 years were 3 times more likely to be 10‐year survivors than those with shorter PFS. Patients receiving no more than two lines of treatment were more likely than multiply treated patients to survive 10 years. Those who achieved at least one CR were more likely to survive 10 years than those who never achieved a CR, although it appeared not to be important whether the CR was in response to first, second or third‐line treatment. Patients who received second‐line treatment were twice as likely to survive 10 years if they received the more effective treatments (FC‐based combinations/SCT).

In multivariate analysis (*n* = 514), the 10‐year survivors were more likely than those who died within 10 years to be aged <70 years, in the good risk group, to have had a long first‐line PFS (>3 years), have received not more than two lines of treatment, achieved at least one CR and to have stage A‐progressive disease. In the subset of patients (*n* = 355) who received second‐line treatment, treatment with FC‐based combinations/SCT was an independent predictor of 10‐year survival [OR 0·40 (0·21–0·76), *P* = 0·005].

Available laboratory markers significantly associated with 10‐year survival in univariate analysis were as follows: (i) markers included in the definition of the risk groups: *IGHV* mutated status; low beta‐2 microglobulin level; absence of deletions of 11q and *TP53*; and (ii) other markers: absence of mutations of *TP53*,* NOTCH1* and *SF3B1*; low expression of CD38, Zap70 and *CLLU1*; and 13q deletion (Table 4). The first 4 markers listed in Table 4 were included in the main multivariate analysis (Table 3) as components of the risk group classification. Further multivariate models were constructed adding each of the other 7 significant variables in Table 4, separately, to the Table 3 variables, including risk group. In subsets including 446 and 361 patients respectively, low expression of CD38 [OR 0·44 (0·25–0·78), *P* = 0·005] and normal *SF3B1* [OR 0·21 (0·07–0·67), *P* = 0·008] were each independent predictors of 10‐year survival, in addition to the variables that were significant in the main multivariate analysis. Inclusion of all variables simultaneously in a multivariate analysis has not been reported here due to a much reduced (*n* = 225) and significantly biased patient subset. Prognostic factor analysis, including all the data from the LRF CLL4 trial, is covered more broadly by the CLL International Prognostic Index project (Bahlo *et al*, 2015).

**Table 3:** Clinical characteristics of patients surviving 10 years or more from randomization

Variable		Trial patients assessable *n* = 733^a^	Survived 10 years *n* = 176 (**24**%^a^)	*P*‐value & Odds ratios^c^ (95% CI)	
				Univariate	Multivariate *n* = 514
Randomized first‐line treatment	Chlorambucil	363^a^	87 (**24** a)	***P* = 1·0**	Not significant
	Fludarabine (F)	184^a^	45 (**24** a)	1·03 (0·68–1·55)	
	F + cyclophosphamide	186^a^	44 (**24** a)	0·98 (0·65–1·49)	
Gender	Female	192	63 (**33**)	**0·001**	Not significant
	Male	541	113 (**21**)	0·54 (0·38–0·78)	
Age group (years)	<60	231	84 (**36**)	**<0·0001**	**<0·0001**
	60–69	273	70 (**26**)	0·60 (0·41–0·88)	0·42 (0·23–0·77)
	70+	229	22 (**10**)	0·19 (0·11–0·31)	0·09 (0·04–0·21)
Disease stage (Binet)	A progressive	181	57 (**31**)	**0·03**	**0·0009**
	B	325	71 (**22**)	0·61 (0·40–0·92)	0·29 (0·15–0·56)
	C	227	48 (**21**)	0·58 (0·37–0·91)	0·59 (0·30–1·14)
Risk group^b^	Good	112	61 (**54**)	**<0·0001**	**<0·0001**
	Intermediate	369	55 (**15**)	0·13 (0·08–0·21)	0·23 (0·13–0·39)
	Poor	33	0 (**0**)	(Good *versus* intermediate/poor)	
Length of first‐line PFS	Long (>3 years)	233	107 (**46**)	**<0·0001**	**<0·0001**
	Short (<3 years)	500	69 (**14**)	0·19 (0·13–0·27)	0·26 (0·15–0·46)
No. of lines of treatment	1	235	72 (**31**)	**<0·0001**	**0·0005**
	2	250	71 (**28**)	0·90 (0·61–1·33)	0·88 (0·47–1·66);
	3 or more	248	33 (**13**)	0·35 (0·22–0·55)	0·25 (0·12–0·55)
Responses to treatment	CR (first‐line)	110	41 (**37**)	**0·0001**	**0·04**
	CR (but not until 2nd/3rd‐line)	68	23 (**34**)	0·86 (0·46–1·62)	2·37 (0·88–6·39)
	No CR	555	112 (**20**)	0·43 (0·27–0·66)	0·81 (0·40–1·62)
Second‐line treatment	FC‐based combinations/SCT	146	47 (**32**)	**<0·0001**	Not included^d^
	All other treatments	352	57 (**16**)	0·41 (0·26–0·64)	

FC‐based combinations/SCT, combination treatments based on fludarabine with cyclophosphamide, or stem cell transplant (including autografts and allografts); CR, complete response; CI, confidence interval; PFS, progression‐free survival.

a 44 overseas patients, who had not reached 10‐year survival by the end of clinical follow‐up, are excluded from this analysis (see Methods section). Thus the percentages shown here do not correspond to those shown in Fig 2A, which include these 44 patients censored at the date of last known contact.

b Poor risk – known *TP53* deletion >10%; intermediate risk – without *TP53* deletion (≤10%) and with at least one of: unmutated *IGHV* genes and/or *IGHV3‐21* usage, 11q deletion, β‐2 microglobulin ≥4 mg/l; good risk – none of the above and mutated *IGHV* genes (Oscier *et al*, 2010).

c The odds ratio for the reference category for each variable = 1·0. *P*‐values are shown in bold and correspond to tests for heterogeneity.

d This variable was not included in the main multivariate analysis because it applied only to the sub‐group who received second‐line treatment.

**Table 4:** Biological, cytogenetic and molecular characteristics of patients surviving 10 years or more from randomization

Variable		No. trial patients assessable	Survived 10 years (%)	*P*‐value^c^
*IGHV* mutation status (cut‐off 98%^a^)	Mutated	205	87 (43)	<0·0001
	Unmutated	321	40 (12)	
beta‐2 microglobulin (cut‐off 4 mg/l^a^)	Low	286	100 (35)	<0·0001
	High	233	26 (11)	
11q deletion	No	456	127 (28)	0·0002
	Yes	116	13 (11)	
*TP53* deletion (cut‐off 10%^a^)	No	531	138 (26)	0·0007
	Yes	33	0 (0)	
*TP53* mutation	No	482	126 (26)	0·0008
	Yes	40	1 (3)	
*NOTCH1* mutation	No	417	108 (26)	0·03
	Yes	45	5 (11)	
*SF3B1* mutation	No	360	107 (30)	0·0004
	Yes	73	7 (10)	
CD38 expression (cut‐off 7%^a^)	Negative	197	85 (43)	<0·0001
	Positive	330	47 (14)	
Zap70 expression (cut‐off 10%^a^)	Negative	241	79 (33)	0·0004
	Positive	233	43 (18)	
*CLLU1* expression (cut‐off RQ 40^b^)	Low	245	79 (32)	0·0001
	High	266	46 (17)	
13q deletion	No	230	40 (17)	0·001
	Yes	342	100 (29)	
trisomy 12	No	481	121 (25)	0·4
	Yes	91	19 (21)	

See also Oscier *et al* (2010) and Oscier *et al* (2013) for multivariate analysis of molecular/laboratory prognostic factors in LRF CLL4.

RQ, real time relative quantification.

a Oscier *et al* (2010).

b Gonzalez *et al* (2013).

c Chi‐squared test.

## Discussion

For patients with CLL requiring treatment and entered into a randomized clinical trial, the ensuing disease course is diverse, ranging from disease progression to first remissions lasting a decade or more. In this study we have focussed on the long‐term outcomes in the LRF CLL4 trial, examining the effect of salvage treatments on survival and identifying some of the characteristics associated with survival of 10 years or more. OS from randomization remained nearly identical in the three trial arms at every point throughout the years, in spite of the significantly longer first‐line PFS in the FC arm. Our main findings suggest that both favourable prognostic markers at randomization as well as effective salvage therapy contributed to 10‐year survival.

The 24% of patients who survived 10 years differed in both their pre‐treatment characteristics and their response to salvage therapy compared to those with poorer outcomes. They were more likely to be younger (<70 years), to have stage A‐progressive rather than stage B or C disease and to have a good‐risk prognostic profile (Oscier *et al*, 2010). The risk factors studied included those factors initially reported (Oscier *et al*, 2010), as well as other, more recently described, gene mutations (Oscier *et al*, 2013) (Table 4). The good risk factors associated with long survival mirror those identified by Rossi *et al* (2013) and, conversely, salvage treatment was ineffective in non‐responders to first‐line FC, two‐thirds of whom had a *TP53* deletion (Fig 2D).

Ten‐year survival from randomization was also associated with longer first‐line PFS, receipt of no more than two lines of treatment, at least one CR in the patient's treatment history, and with the type of salvage treatment received. The importance of a long PFS and achieving a (clinical) CR are consistent with the findings both of Tam *et al* (2014), who showed that patients treated with FCR with a first‐line PFS longer than 3 years survive better in the long‐term than those with PFS <3 years, and also of the VISTA (Velcade^®^ as Initial Standard Therapy in Multiple Myeloma) trial in multiple myeloma, in which a CR either after initial therapy or after relapse was equally associated with longer OS (Harousseau *et al*, 2010).

Patients treated less intensively at first line, particularly with chlorambucil, had improved CR rates when treated at second and third line, and good responses to salvage treatment were associated with longer OS. Second‐line treatment with FC‐based combinations or SCT was given to a third of re‐treated patients in each arm and, when compared to all other salvage therapies, led to significantly improved OS after progression. Use of these more effective second‐line therapies was also a strong predictor of 10‐year survival (Table 3, Fig 3C).

The inclusion of rituximab in FC‐based combinations was beneficial as part of the salvage therapy, consistent with previous studies on the use of FCR and FCMR (fludarabine, cyclophosphamide, mitoxantrone, rituximab) in relapsed CLL (Wierda *et al*, 2005; Robak *et al*, 2010; Hillmen *et al*, 2011; Tam *et al*, 2014). Interestingly, 19 of the 24 patients in this study who underwent SCT had an initial PFS shorter than 3 years. The curative potential of SCT for some patients has been well documented by data from the European Group for Blood and Marrow Transplantation registry, which showed 35% OS at 10 years (Van Gelder *et al*, 2014). The median age of patients was 55 years, similar to the SCT group in our own series (median 54 years).

Use of the most effective salvage therapies, FC‐based combinations or SCT, was more important for long OS in patients who previously received fludarabine, and particularly FC, than in patients who initially received chlorambucil (Fig 4). This is likely to reflect the higher percentage of patients in the chlorambucil arm who received a more effective salvage therapy compared to their first‐line therapy (including, for example, single‐agent fludarabine), while the options for a more effective salvage therapy were fewer for patients relapsing after FC. The genomic landscape of patients before treatment is variable and often comprises a dominant clone together with one or more sub‐clones. Clonal evolution is more common after treatment than in untreated CLL (Landau *et al*, 2013) and small sub‐clones (e.g. *TP53*‐mutated sub‐clones not detected initially by conventional methods) can become predominant after relapse (Rossi *et al*, 2014; Sutton & Rosenquist, 2015). Further longitudinal genomic studies are required to determine whether therapies such as FC, which induce a powerful DNA damage response, select for small drug resistant clones that impair response to subsequent chemo or chemo‐immunotherapy (Rosenwald *et al*, 2004; Landau *et al*, 2013; Rossi *et al*, 2014).

In summary, this study demonstrates the importance and clinical relevance of long‐term follow‐up in clinical trials of patients with CLL. It shows that even those patients who have received less effective initial treatments, such as chlorambucil, can be rescued with more effective salvage therapy, provided a good response can be achieved. We have confirmed the benefit of FC‐based combinations as salvage therapy, particularly those including rituximab, as already shown by others. We also confirm the value of SCT in a small group of younger individuals, especially those with short PFS after first‐line treatment. Long‐term survival was greatly influenced by well‐known clinical characteristics (age, stage, response, length of PFS) and by prognostic risk groups defined by laboratory and molecular markers. While it seems likely that the outlook for patients with CLL will be transformed by the use of novel agents, long‐term follow‐up in clinical trials which incorporate both established and novel biomarkers will continue to be needed to determine the true long‐term benefit of these agents. Our findings suggest that, as with the earlier generation of drugs used in this trial, the challenge with the newer antibodies, BCL2 antagonists and BCR kinase inhibitors will be to find treatments effective against the more resistant clones emerging after relapse, so that the initial strong performance seen with these agents can translate into improved OS compared to that of patients receiving the more established treatments. A CR obtained with second‐ or third‐line agents can prolong survival.

## Author contributions

DC was the principal investigator and takes primary responsibility for the paper; ME performed the analyses; RW oversaw the preparation of the data and gave statistical advice and support; DO did the core research on prognostic factors in this trial; ME, DO and DC wrote the paper. The authors report no potential conflicts of interest.
